# Precipitation and Neutralization of Heparin from Different Sources by Protamine Sulfate

**DOI:** 10.3390/ph10030059

**Published:** 2017-07-02

**Authors:** John Hogwood, Barbara Mulloy, Elaine Gray

**Affiliations:** 1 National Institute for Biological Standards and Control (NIBSC), Blanche Lane, South Mimms, Herts EN6 3QG, UK; elaine.gray@nibsc.org; 2 Institute for Pharmaceutical Sciences, King’s College London, 10 Stamford Street, London SE1 9HN, UK; barbara.mulloy@kcl.ac.uk

**Keywords:** heparin, protamine sulfate, neutralization

## Abstract

Current therapeutic unfractionated heparin available in Europe and US is of porcine mucosal origin. There is now interest, specifically in the US, to use bovine mucosa as an additional source for the production of heparin. The anticoagulant action of heparin can be neutralized by protamine sulfate, and in this study the ability of protamine to bind and neutralize the anticoagulant activities of heparin from porcine mucosa, bovine mucosa and bovine lung were assessed. Protamine sulfate was able to bind and precipitate similar amounts of heparins from different sources on a mass basis. However, differential amounts of anticoagulant activities were neutralized by protamine sulfate, with neutralization of porcine mucosa more effective than for bovine lung and bovine mucosa. For all heparins, potentiation of thrombin inhibition by antithrombin and heparin cofactor II was preferentially neutralized over antithrombin-mediated inhibition of factor Xa or plasma clotting time. Whole blood thromboelastography showed that neutralization by protamine sulfate was more effective than the antithrombin dependent thrombin inhibition assays indicated. While there was no absolute correlation between average or peak molecular weight of heparin samples and neutralization of anticoagulant activity, correlation was observed between proportions of material with high affinity to antithrombin, specific activities and neutralization of activity.

## 1. Introduction

Contamination of porcine heparin with oversulfated chondroitin sulfate in 2007/2008 [1] led to rapid revisions to pharmacopoeial monographs. The principal changes made to the United States Pharmacopeia (USP) and European Pharmacopeia (EP) monographs for heparin included specifying the source as porcine intestinal mucosa, adoption of a purified reagent assay based on antithrombin inhibition of thrombin for potency determination and a specific activity of not less than 180 IU/mg [2]. Furthermore, the contamination issue highlighted the primary reliance on a single source for clinical heparin, porcine mucosa, in Europe and the US. There is now interest in diversifying the supply of heparin, with bovine mucosa suggested as an alternative source [3].

Heparin is a complex heterogeneous and polydisperse anionic polysaccharide, consisting of alternating glucosamine and uronic acid monosaccharide residues [4]. Its primary use is as an anticoagulant and antithrombotic agent for surgery and extracorporeal circuits [5]. Heparin is the anticoagulant of choice for these indications as it can be readily reversed by protamine sulfate [6], an arginine rich basic protein [7]. The use of protamine sulfate is not without some risk [8], and there has been interest in alternative compounds which can neutralize heparin activity [9,10,11,12]. However, such investigations have been limited in scope, primarily involving in vitro experimentation. In an animal model [10], a synthetic compound with lower immunogenicity was found to be able to neutralize heparin comparably to protamine. Despite the interest in alternatives to protamine sulfate, until clinical evaluations are carried out it will remain the heparin neutralization agent of choice.

Porcine intestinal mucosa (PM) heparins have been used extensively for over 50 years, with gradual improvements in quality and specific activity [13]. Bovine heparins, whilst clinically used in South America, have not been subjected to extensive study until recently [14,15]. Primary observations are that bovine and porcine heparins have structural differences [14] such as lower weight average molecular weight for bovine lung (BL) heparin, and a lower proportion of 6-*O*-sulfo groups in bovine intestinal mucosa (BM) heparin. Bovine heparins also have lower specific activities [16], with values consistently below the new minimum monograph potency requirement. The lower specific activity would mean that greater amounts by mass of bovine heparin would be required to achieve the same dose in units of activity.

There is limited information on the ability of protamine sulfate to neutralize the activity of heparin from different sources. Recent interest in the interaction between protamine sulfate and heparin has centered on the possible immunogenic nature of large macromolecular protamine–heparin complexes [17]. Some previous work has shown that high molecular weight heparins have a higher affinity for protamine [18,19], with these studies carried out with porcine unfractionated or low molecular weight heparins. Independent of molecular weight, protamine sulfate has also been shown to neutralize more effectively antithrombin dependent inhibition of thrombin than the antithrombin inhibition of factor Xa [19]. The clinical dosage of protamine sulfate as recommended by the manufacturers is related to the pharmacopoeial methods for bioidentity of protamine sulfate which specify that 1 mg of protamine should precipitate not less than 100 international units (IU) of heparin reference standards. However, it is not clear how the precipitation of heparin relates to neutralization of anticoagulant activity of heparin.

The main objective of this study was to investigate the relationship between precipitation and neutralization of porcine and bovine heparins with protamine sulfate. Accurate anticoagulant activities were determined for a range of PM, BM and BL heparins and used to ascertain whether the units of heparin as measured by heparin/protamine sulfate precipitation related to neutralization of activity. The results from the current study indicate that there are differences in the precipitation and neutralization of biological activity of the heparins by protamine sulfate, and that neutralization of anticoagulant activities by the same amount of protamine sulfate is different for heparin from different sources.

## 2. Results

### 2.1. Anticoagulant Activity of Heparin Samples

**Figure 1:**
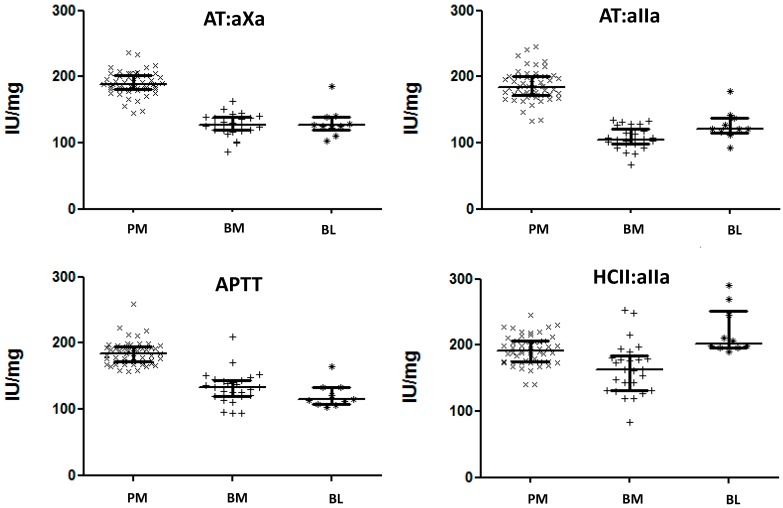
Scatter Plots of specific activities, and interquartile range for porcine intestinal mucosa (PM), bovine intestinal mucosa (BM) and bovine lung (BL) heparin samples. Activities have been calculated relative to the 6th International standard for Unfractionated Heparin (porcine heparin) using parallel line bioassay model for all assays. All samples compared validly to the standard, passing the ANOVA criteria for parallelism and linearity. AT:aXa—antithrombin dependent anti-Xa; AT:aIIa—antithrombin dependent anti-IIa, APTT—plasma APTT assay; HCII:aIIa—heparin cofactor II dependent anti-IIa.

The anticoagulant activities were estimated against the 6th International Standard for Unfractionated Heparin (07/328, NIBSC, South Mimms, UK), a PM heparin. All activity assays: antithrombin-dependent anti-Xa (AT:aXa), antithrombin-dependent anti-IIa (AT:aIIa), human plasma anticoagulant activated thromboplastin time (APTT) and heparin cofactor II dependent anti-IIa (HCII:aIIa) were valid by parallelism and linearity criteria using the statistical package, Combistats 5.0 (EDQM, Strasbourg, France). The activities of fifty-two PM heparins (Figure 1) were similar (AT:aXa 190, AT:aIIa 186, APTT 185, HCII:aIIa 193 IU/mg) irrespective of the method used (ANOVA, *p* = 0.23). For twenty-six batches of BM heparin samples, there was significant difference between the different assay methods (ANOVA *p* < 0.0001). However, there was no significant difference between results for AT:aXa (127 IU/mg) and APTT (133 IU/mg) activities when considered independently of the other assay methods (*p* > 0.01). AT:aIIa potencies (107 IU/mg) were lower and the HCII:aIIa activities (165 IU/mg) were higher than AT:aXa and APTT estimates. For ten batches of bovine lung heparin, the AT:aXa, AT:aIIa and APTT (130, 127, 121 IU/mg respectively) activities were similar (ANOVA, *p* > 0.01), but HCII:aIIa (220 IU/mg) activity was significantly higher than all other activities (ANOVA, *p* < 0.0001).

With the exception of HCII:aIIa for bovine lung heparin, PM heparin had higher anticoagulant activities (Figure 1) than BM and BL heparins. BM and BL heparins had similar activities by AT:aXa (127 to 131 IU/mg), but different activities by AT:aIIa (107 to 127 IU/mg) and by APTT (133 to 121 IU/mg). The higher average HCII:aIIa activity of BL heparin (220 IU/mg) compared to PM heparin (193 IU/mg) and BM heparin (165 IU/mg) was influenced by three out of 10 BL heparin samples having exceptionally high activities, thus raising the overall average HCII:aIIa value. When the median HCII:aIIa activity is compared, BL heparin (203 IU/mg) is only slightly higher than PM heparin (191 IU/mg).

### 2.2. Protamine Sulfate Precipitation Assay

**Figure 2:**
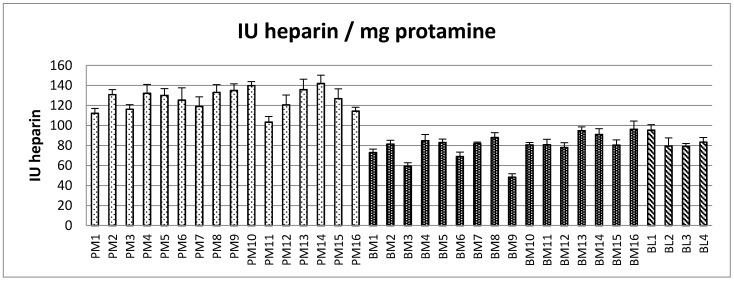
Amount of heparin in International Units (anti-IIa) that is required to precipitate 1 mg of protamine sulfate, estimated according to the European Pharmacopeia assay for protamine sulfate. (error bars = standard deviation, *n* = 9).

The European Pharmacopeia (EP) monograph states that 1 mg of protamine sulfate precipitates not less than 100 IU of Biological Reference Preparation (BRP) heparin standard. A selection of 16 PM, 16 BM and 4 BL heparin samples were used in the precipitation assay (Figure 2). For this set of experiments, the quantity of heparin (based on AT:aIIa unitage) needed to precipitate 1 mg protamine sulfate was measured. Broadly, greater amounts of PM heparins by activity (average of 126 AT:aIIa IU, ranging 103–142 AT:aIIa IU) were required to precipitate protamine sulfate than BM heparin (an average of 79 IU AT:aIIa, ranging from 48–96 AT:aIIa IU) and BL heparins (an average of 84, ranging from 79–95 AT:aIIa IU).

**Figure 3:**
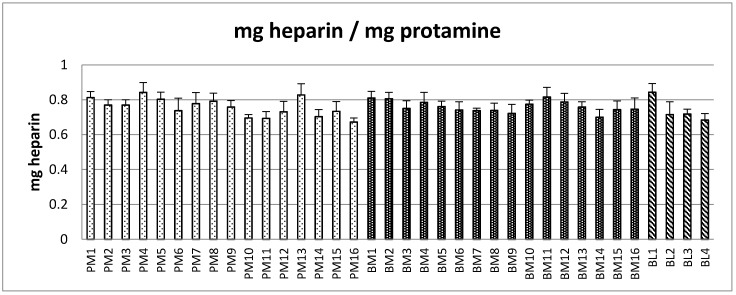
Amount of heparin in mg (calculated from anti-IIa specific activity) that is required to precipitate out 1 mg of protamine sulfate, estimated according to the European Pharmacopeia assay for protamine sulfate. (error bars = standard deviation, *n* = 9).

Based on the AT:aIIa specific activity estimated, the corresponding amounts of heparin in terms of mass were recalculated from the amount in IU. The heparin by mass which precipitated 1 mg of protamine sulfate is shown in Figure 3. By contrast, the average mass concentration required to precipitate the same amount of protamine sulfate was the same for all the heparins, PM 0.76 mg, BM 0.76 mg and BM 0.74 mg. Table 1 shows that on an IU basis PM and BM are clearly different (*p* < 0.001), but on an mg basis the same amount of heparin was needed to precipitate 1 mg of protamine sulfate.

### 2.3. Neutralization of Heparin Activity

**Figure 4:**
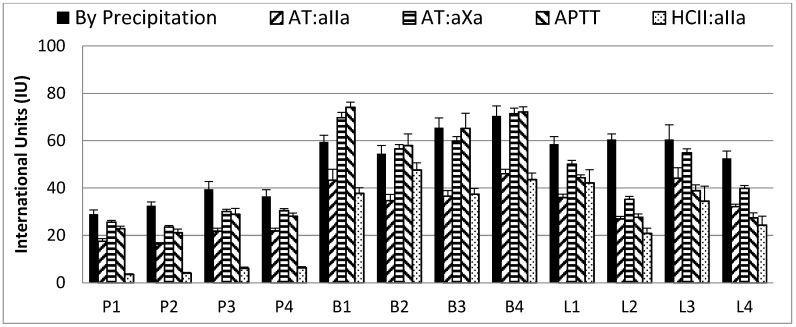
Remaining activity from precipitation and neutralization assays for each heparin sample. Potency shown above has been calculated from mixture of 100 IU/mL heparin with 0.5 mg/mL protamine sulfate, precipitation assay value has been calculated from the protamine sulfate assay, AT:aIIa, AT:aXa, APTT and HCII:aIIa are residual potency estimations. AT:aIIa—antithrombin dependent anti-IIa, AT:aXa—antithrombin dependent anti-Xa, APTT—plasma APTT clotting assay, HCII:aIIa—Heparin Cofactor II dependent anti-IIa. Error bars are SD for precipitation assay, and upper 95% confidence limit for other assays.

Four samples each of PM, BM and BL heparin, spanning the range of specific activities by AT:aIIa assays, were investigated further (Table 2) for neutralization of activities. With a fixed amount of heparin mixed with a fixed concentration of protamine sulfate, the remaining amount of heparin by precipitation and by anticoagulant activity was estimated (Figure 4).

In general, for all the samples, protamine sulfate was able to neutralize more biological activity than the precipitation assays indicated. For PM heparin, the anticoagulant activity through HCII was more readily neutralized than antithrombin dependent activity or plasma based activity (Figure 4). For example, for sample P1, the remaining activities by AT:aIIa, AT:aXa, APTT and HCII:aIIa were 17.5, 25.6, 28.5 and 3.5 IU respectively. The ranking order for neutralization was HCII:aIIa > AT:aIIa > AT:aXa = APTT across the four samples. For BM heparin, HCII and AT:aIIa were neutralized to a similar extent, and AT:aXa and APTT neutralization were also similar to each other. For example, for sample B1 the remaining activities by AT:aIIa, AT:aXa, APTT and HCII:aIIa were 43.4, 69.7, 74.1 and 37.7 IU respectively. The ranking order for neutralization was HCII:aIIa = AT:aIIa > AT:aXa = APTT. The BL heparin ranking order for neutralization was HCII:aIIa > AT: aIIa > APTT > AT:aXa. When comparing the differences between neutralization and precipitation, only the BM samples had values which were similar. This is illustrated by the remaining level of anticoagulant activity in BM samples by AT:aXa (69.7, 56.5, 59.8, 71.4 IU) and APTT (74.1, 57.9, 65.2, 72.0 IU) were similar to the precipitation values (59.5, 54.5, 65.5, 70.5 IU).

The average amount of heparin which protamine sulfate can precipitate or neutralize is shown in Table 3. Protamine sulfate neutralized more activity in BM, 123 AT:aIIa IU/mg, than indicated by precipitation, 75 IU/mg, a 64% difference. The difference between neutralization and precipitation for BL heparin was 56%, 131 and 84 IU/mg respectively. Neutralization of PM heparin by protamine sulfate was also higher than precipitation indicated, 161 IU/mg from 131 IU/mg. For all heparins, neutralization of anticoagulant activity measured by AT:aIIa was higher than indicated by precipitation. Calculation using the specific activities of the heparins for a mass/mass interaction between the heparins and protamine sulfate showed that by neutralization the amount of heparin required was no longer similar as shown by the precipitation assay.

### 2.4. Thromboelastography

**Figure 5:**
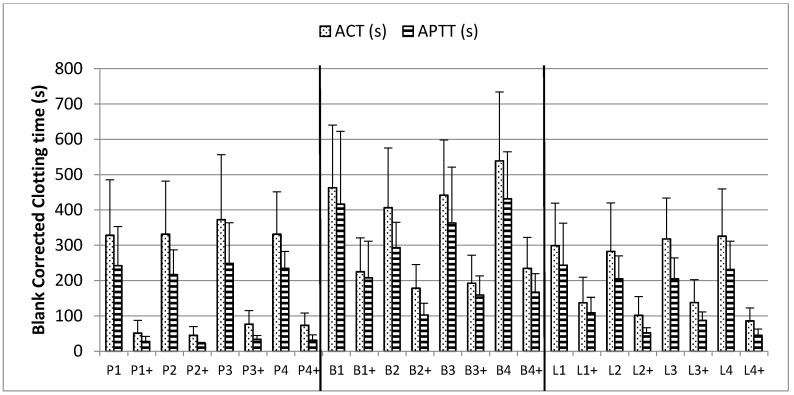
Activated clotting from thromboelastography and clotting times from APTT assay of heparin samples at 1 IU/mL without (P1, B1, B2 etc.) or with 0.05 mg/mL protamine sulfate added (P1+, B1+, B2+ etc.) in whole blood. Data shown are the blank corrected clotting times averaged from six individual blood donors, a 0 s time would indicate that all heparin present has been neutralized. (*n* = 6, error bars = SDs).

The thromboelastography activated clotting time (ACT) and whole blood APTT clotting time is shown in Figure 5. For both ACT and APTT, the clotting times were different for each donor (*n* = 6) with and without heparin (data not shown). The reported clotting times for each heparin sample are the averaged clotting times from all donors and have each been blank corrected (heparin clotting times minus heparin-free clotting time).

The same amount by AT:aIIa activity of each heparin was used to prepare the samples using each donor’s blood. The ACT measured was significantly different between the samples, PM to BM *p* = 0.002, PM to BL *p* = 0.046, and BM to BL *p* = 0.001. In the APTT assays, there was a difference between the PM and BM (*p* = 0.004), the BM and BL (*p* = 0.001) but not between PM and BL (*p* = 0.157).

The addition of protamine sulfate reduced the anticoagulant activity in all heparin samples. The reduction in clotting times was more pronounced for PM than BM or BL (average ACTs decreased from 341 to 62s, 462 to 207s and 306 to 116s respectively). The order of neutralization was PM, BL and BM, the same as that seen in the anticoagulant assays (Figure 4).

### 2.5. Molecular Weight

Molecular weights for the heparin samples are shown in Table 4. Except for one BM sample, B2, porcine heparins had higher molecular weights than the bovine heparin samples. The peak molecular weight, number average and weight average molecular weights for the bovine lung samples were lower than BM heparin. The polydispersity for all the heparins was comparable for each heparin type, ranging from 1.20 to 1.29.

### 2.6. Antithrombin Titration Assay

**Figure 6:**
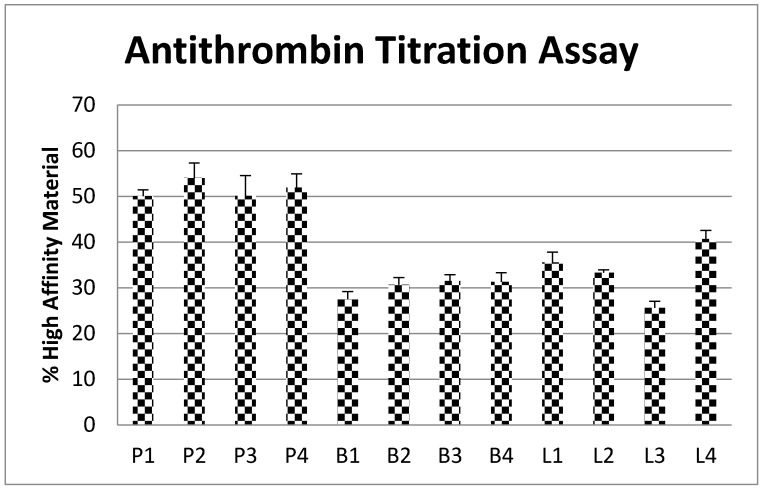
Antithrombin fluorescent titration assay with % high affinity antithrombin binding material within each heparin preparations. High affinity material calculated using the number average molecular weight Mn for each heparin and the concentration of heparin required to titrate antithrombin to generate a maximum change in fluorescent signal. (*n* = 4, error bars = SDs).

The amount of antithrombin high affinity binding material in each heparin sample is shown in Figure 6. The PM samples had a similar quantity of high affinity material to each other, with the average level 52%. The BM heparins had an average 30% high affinity material with little difference between the samples. The level of high affinity material in BL heparin was more variable, ranging from 25 to 40% and an average of 33%. The amount of antithrombin binding high affinity material was higher in PM heparin than both BM and BL (about 70% and 60% more respectively).

## 3. Discussion

In this study, we show that the specific activities of PM, BM and BL heparins are different. The potency labelling assay, antithrombin dependent inhibition of thrombin (AT:aIIa), gave much lower values for both BM heparin and BL heparin samples when compared to PM heparin which confirms previous observations [14,20]. Of note, all the samples tested gave statistically valid results for all the activity assays against the 6th International Standard for Unfractionated Heparin. This indicates that the International Standard, a PM heparin, is a suitable potency standard not only for PM heparin but also for BM and BL heparins.

The pharmacopoeial specifications for PM heparin are for potency, a specific activity not less than 180 IU/mg or USP U/mg, and for identity a ratio of AT:aIIa/AT:aXa values within 0.9 to 1.1. All PM heparins passed the identity criterion and most passed the specific activity requirement of >180 IU/mg. PM heparins also gave anticoagulant activities by APTT and HCII:aIIa assays that were similar to the antithrombin dependent assays. Both BM and BL heparins have lower specific activity than the >180 IU/mg required for PM heparin. Furthermore, BM heparin had an AT:aIIa/AT:aXa ratio that was outside the 0.9–1.1 criteria, with a calculated average of 1.2. It has been suggested that it will be unlikely for manufacturers to achieve the 180 IU/mg criteria for bovine heparin samples [20] and the data here support this. It should be noted that the lower specific activities of the bovine heparins indicate that more heparin in terms of mass would be required to achieve a similar anticoagulant effect to PM heparin.

The reversal of PM heparin post-operatively by protamine sulfate has been well established [21]. Protamine sulfate quality is assessed by a straightforward titration assay with heparin, with the minimal requirement that 1 mg precipitates not less than 100 IU of the pharmacopoeial reference preparations which are porcine heparins. However, it is unclear whether 1 mg protamine sulfate will precipitate as much as 100 IU of BM or BL heparin. The current study has shown that 1 mg protamine sulfate precipitated less than 100 IU of bovine heparins.

Irrespective of the specific activities of the samples, the amount of heparin in mass needed to precipitate a given quantity of protamine sulfate is the same for all heparins. In terms of activity units however, the ability of heparin to precipitate protamine sulfate was linked to the specific activities of the various heparins. A lower specific activity results in an increased concentration in units of heparin being required to precipitate a given amount of protamine sulfate.

The precipitation assay for protamine sulfate assesses the quality of protamine sulfate (a bio-identity test) and does not provide an indication of its ability to neutralize heparin activity. The use of a fixed activity of heparin for all preparations along with a fixed amount of protamine sulfate allowed for the estimation of the amount of activity neutralized. For all samples, the ability of protamine sulfate to neutralize activity was higher than would be predicted from the amount of heparin that can be precipitated. As expected, the use of fixed activity with a fixed amount of protamine showed that PM heparins, with have higher specific activities, were more effectively neutralized than BM and BL heparins.

Broadly, the thrombin based assays were more readily neutralized which may be due to the extended heparin chain length being more critical for these methods [22]. However, despite PM heparins having higher molecular weight than the BM and BL heparins, there is no significant correlation between the neutralization of activity and the molecular weight profiles of the heparin tested. The anti-Xa and plasma APTT anticoagulant activities were neutralized to a similar degree. The interaction between negatively charged heparin and positively charged protamine sulfate will favour the longer oligosaccharides in heparin [19] and as shown here influence antithrombin dependent thrombin inhibition activity which requires a minimum heparin molecular weight of about 5400 [16].

Clinically protamine sulfate will be administered by perfusion in order to neutralize heparin, and neutralization can be assessed using thromboelastography. This point-of-care technique is used with patient whole blood, where success of heparin neutralization is assessed by observing the time at which responses return to baseline. In this study, a controlled approach with the same amount of heparin, 1 IU anti-IIa, spiked into whole blood was used. We showed that the clotting times for each heparin type were different. In addition, at the same potency (AT:aIIa units), BM heparin gave longer activated clotting times than both PM and BL. The addition of protamine sulfate gave a reduction in clotting times for all samples, with the greatest reduction in PM, then BL and finally BM. This matched the profile of responses for the anticoagulant assays.

Fluorescence titration with antithrombin showed that PM heparin has a higher proportion of high affinity binding material than both BL and BM heparins. This correlates with the higher specific activity seen for PM heparin, and also explains the greater effectiveness of protamine sulfate at neutralizing the activity of PM heparin. Whilst having similar proportions of high affinity binding material and antithrombin dependent inhibition, BM and BL heparins show some differences in neutralization profiles. Neutralization of BL heparin is slightly greater than for BM heparin, which coincides with the slightly higher anti-IIa specific activity for bovine lung heparin.

For therapeutic purposes, an increase in mass of BM or BL heparin would be required to achieve the same therapeutic dose in International or USP Units of PM heparin. Based on the results of this study, it would be important to assess whether the current regimen of neutralization by protamine sulfate would need to be revised.

## 4. Materials and Methods

### 4.1. Materials

PM, BM and BL heparin samples were from the NIBSC archive of heparin samples, donated by several manufacturers. All heparin samples were subjected to a freeze dry step for 24 h prior to testing. Protamine sulfate was from Wockhardt UK Ltd, Wrexham, UK.

### 4.2. Anticoagulant Assays

Anticoagulant activity was estimated against the 6th International Standard for Unfractionated Heparin (07/328, NIBSC, South Mimms, UK). Antithrombin dependent anti-Xa (AT:aXa) and anti-IIa (AT:aIIa) activities were carried out in accordance to the USP Monograph (USP 34 NF 29). Briefly, heparin samples are mixed with purified antithrombin (NIBSC, South Mimms, UK) prior to the addition of thrombin (FIIa) (NIBSC, South Mimms, UK) or factor Xa (Diagnostic Reagents, Thame, UK). After incubation a specific chromogenic substrate (S2238 and S2765 respectively, Werfen Ltd, Warrington, UK) was added, and the colour developed is inversely proportional to the amount of heparin. APTT assay was carried out in accordance with the European Monograph (01/2008:20705) but with human pooled plasma used instead of sheep plasma. Briefly, heparin samples are mixed with plasma to which an APTT regent (APTT-SP, Werfen Ltd, Warrington, UK) is added. After incubation, calcium chloride was added and the clotting time measured, which is directly proportional to the amount of heparin present. Heparin cofactor II dependent activity (HCII:aIIa) was measured by substitution of antithrombin with heparin cofactor II (Enzyme Research, Swansea, UK) in the USP anti-IIa method, and prolongation of the incubation steps from one to seven minutes [23].

All activities were estimated and assessed for statistical validity using the parallel line model in the statistics package Combistats 5.0 (EDQM, Strasbourg, France).

### 4.3. Protamine Sulfate Precipitation Assay

The titration method, as described in the European Monograph for protamine sulfate (01/2017:0569), was adapted for use with the heparin samples. This method as described is a titration assay for protamine sulfate, where the requirement is that 1 mg of protamine sulfate precipitates not less than 100 IU of heparin sodium BRP. The method was adapted to allow for the calculation of heparin IU which precipitates 1 mg of protamine sulfate. Briefly, increasing volumes of heparin were added to a fixed concentration of protamine sulfate until a sharp increase in optical density was observed (wavelength is not critical). The sharp increase was then used to calculate the amount of heparin which has precipitated protamine sulfate.

### 4.4. Heparin/Protamine Sulfate Complex

Several heparin samples, 4 each of PM, BM and BL, were diluted to 200 anti-IIa IU/mL and either mixed with an equal volume of 1 mg/mL protamine sulfate or deionised water. Anticoagulant activity, as above, was then measured for all prepared samples.

### 4.5. Whole Blood Assays

Citrated whole blood was obtained through the National Blood and Transfusion Service (NBTS, New Brunswick, NJ, USA) and used to dilute samples of heparin (100 IU/mL) and heparin/protamine sulfate (100 IU/mL, 0.5 mg/mL) as described in Section 4.4. The clotting time of each sample, diluted 100 fold in whole blood, was measured by Activated Partial Thromboplastin Time (APTT) in a KC4 coagulometer. The activated clotting time was measured using thromboelastography (TEG, Haemonetics, Coventry, UK) with tissue factor (14/302, NIBSC, South Mimms, UK) and calcium chloride as the activating reagent.

### 4.6. Molecular Weights

Molecular weights for all samples were determined using size-exclusion chromatography with the USP Heparin Sodium Molecular Weight Calibrant RS (US Pharmacopeial Convention, MD, USA) as the broad standard calibrant as previously described [24].

### 4.7. Antithrombin Fluorescent Titration

The binding of heparin to antithrombin was assessed using a fluorescence titration assay [25]. This method, which relies on a change in intrinsic fluorescence of antithrombin when bound to heparin, was adapted for use in a microtitre plate. All materials were diluted in 50 mM TRIS, 150 mM NaCl, 10 mM EDTA, 0.05% Tween-20 pH 7.4 buffer (all reagents from, Sigma, UK). A fixed concentration of antithrombin (NIBSC, South Mimms, UK) was placed into each well in a microplate, and increasing concentrations of heparin, 0 to 100 µg/mL, or fondaparinux (USP Fondaparinux Sodium for Assay, USP, Rockville, MD, USA), 0 to 6 µg/mL were added across wells. The increase in fluorescence (λ_EX_ 280 nm, λ_EM_ 340 nm) was measured and using the equation: ΔF_[H]_ = (F_[H]_–F0)/(Fm–F0), where F_[H]_ is measured fluorescence at a concentration of heparin [H], F0 is basal fluorescence and Fm is maximum fluorescence, a chart was plotted of ΔF_[H]_ vs heparin concentration. From this chart, the amount of high affinity material in heparin which binds to antithrombin was calculated as the heparin concentration value at ΔF_[H]_ = Fm on an extrapolated straight line fitted to the first five heparin concentration points. The percentage high affinity material was then calculated using the concentration of antithrombin, where the molar concentration was estimated based on the assumption of 1:1 binding between antithrombin and fondaparinux, and molar concentration of heparin, calculated using number average molecular weight.

## Figures and Tables

**Table 1:** Protamine Sulfate precipitation results for heparin samples by IU/mg and mg/mg. Results show the mean and range for each heparin type for IU (anti-IIa) to mg protamine and recalculated for mg (anti-IIa) to mg protamine.

	PM (*n* = 16)	BM (*n* = 16)	BL (*n* = 4)	*p*-Values
**IU/mg PS Mean (range)**	126 (103–142)	79.4 (48.4–96.2)	84.3 (79.0–95.3)	PM–BM < 0.001; PM–BL < 0.001; BM–BL = 0.233
**mg/mg PS Mean (range)**	0.76 (0.67–0.84)	0.76 (0.70–0.82)	0.74 (0.68–0.84)	PM–BM = 0.403; PM–BL = 0.289; BM–BL = 0.188

**Table 2:** Selected heparins (four each of PM, BM and BL heparins) IU (AT:aIIa) required to precipitate 1 mg protamine sulfate. Samples here are not PM 1–4 or BM 1–4 as indicated in Figure 2 and Figure 3.

	Sample Code and IU Heparin/mg Protamine			
PM	P1	P2	P3	P4
	142	135	121	127
BM	B1	B2	B3	B4
	81	91	69	59
BL	L1	L2	L3	L4
	81	79	79	95

**Table 3:** Average amount of heparin which 1 mg of protamine sulfate can precipitate or neutralize. (±SDs).

	By EP Precipitation Assay		By Neutralization of Biological Activity	
**Ave (*n* = 4)**	IU anti-IIa/mg protamine	mg heparin/mg protamine	IU anti-IIa/mg protamine	mg heparin/mg protamine
**PM**	131 (±9)	0.73 (±0.02)	161 (±6)	0.90 (±0.06)
**BM**	75 (±14)	0.75 (±0.04)	123 (±9)	1.26 (±0.19)
**BL**	84 (±8)	0.74 (±0.07)	131 (±15)	1.16 (±0.15)

**Table 4:** Molecular Weight of heparin samples, determined against the USP Unfractionated Heparin Molecular Weight Standard, results are average from two determinations. Mp = peak molecular weight, Mn = number average molecular weight, Mw weight average molecular weight, PD = polydispersity.

		Mp	Mn	Mw	PD	% < 8 kDa
Porcine Mucosa	P1	15,800	13,820	17,870	1.29	8.7
	P2	16,110	13,900	17,290	1.24	7.7
	P3	16,320	14,140	17,390	1.23	6.5
	P4	15,080	13,080	15,720	1.2	8.4
Bovine Mucosa	B1	13,230	11,660	14,750	1.27	15.5
	B2	16,140	14,980	17,950	1.2	4.4
	B3	14,130	12,920	15,920	1.23	9.4
	B4	13,940	12,670	15,430	1.22	9.8
Bovine Lung	L1	9700	11,280	14,450	1.28	18.1
	L2	8770	10,570	13,380	1.27	22.7
	L3	8810	9740	12,160	1.25	28
	L4	9720	11,310	14,360	1.27	17.5
